# Application of Molecularly Imprinted Polymers (MIP) and Magnetic Molecularly Imprinted Polymers (mag-MIP) to Selective Analysis of Quercetin in Flowing Atmospheric-Pressure Afterglow Mass Spectrometry (FAPA-MS) and in Electrospray Ionization Mass Spectrometry (ESI-MS)

**DOI:** 10.3390/molecules24132364

**Published:** 2019-06-26

**Authors:** Maria Guć, Grzegorz Schroeder

**Affiliations:** Faculty of Chemistry, Adam Mickiewicz University in Poznań, Uniwersytetu Poznańskiego 8, 61-614 Poznań, Poland

**Keywords:** magnetic molecularly imprinted polymer (mag-MIP), molecularly imprinted polymer (MIP), quercetin, mass spectrometry (MS), ESI-MS, FAPA-MS

## Abstract

Molecularly imprinted polymer (MIP) and magnetic molecularly imprinted polymer (mag-MIP) for solid extraction and pre-concentration of quercetin have been successfully prepared by thermal polymerization method using quercetin (Q) as a template, acrylamide (AA) as a functional monomer, and ethylene glycol dimethacrylate (EGDMA) as a cross-linking agent. The MIP and mag-MIP were successfully applied in analysis of quercetin by mass spectrometry (MS) methods. To perform ambient plasma ionization experiments, a setup consisting of the heated crucible, a flowing atmospheric-pressure afterglow (FAPA) plasma ion source, and amaZon SL ion trap (Bruker, Bremen, Germany) was used. The heated crucible with programmable temperature allowed desorption of the analytes from MIPs structure which resulted in their direct introduction into the ion stream. The results of Q-MIP/Q-mag-MIP and FAPA-MS measurements were compared with those of the analysis of quercetin by the ESI-MS method without extractions and pre-concentration of analytes on polymers. Limits of detection (LOD) for quercetin solutions in both positive and negative ESI-MS were established at 10^−8^ M and 10^−7^ M, respectively. The linearity (R^2^ = 0.9999) of the proposed analytical procedure for quercetin determination in positive ions was provided in the range between 10^−4^ M and 10^−7^ M. Moreover, the same parameters were established for FAPA-MS in positive ions, reaching LOD at 0.005 mg/gMIP and the linearity of the method in the range of 0.015–0.075 mg/gMIP with the correlation coefficient value R^2^ = 0.9850.

## 1. Introduction

Reduction of the stages of sample preparation for quantitative and qualitative determination of analytes is one of the key tasks of contemporary analytical chemistry. One of the approaches proposed is based on the use of selective methods of extraction and significant concentration of the analyte on solid supports in combination with mass spectrometry as the method of analyte detection [1]. Recently, much progress has been made thanks to the method of molecular imprint technology (MIT). The technology of production of molecularly imprinted polymer (MIP) [2,3,4,5,6,7,8,9,10] and magnetic molecular imprinted polymer (mag-MIP) [11] is based on the formation of complexes between the analyte/template/guest molecule and the functional monomer/host molecule. The analyte-monomer complex is then subjected to polymerization initiated by temperature or irradiation (thermal or photoinitiation) during which in the presence of a cross-linking agent, a three-dimensional polymer structure is formed. At the subsequent stage, the template is removed from the polymer structure obtained, leaving empty cavities capable of binding the template molecules as they are complementary to these template molecules in size, shape and functional group positions [12,13,14]. The final properties of polymer scavengers depend on a number of parameters, including the types of monomers chosen, type of their interactions with the template, quantitative ratio of the monomer to the template, conditions of polymerization and the way of removing the template from the polymer (*Soxhlet extraction, dialysis, thermal desorption*). The polymers obtained are chemically stable, water insoluble, which permits their multiple uses in chemical analysis, and their synthesis process is relatively simple, reproducible and cheap. The only limitation of this method in chemical analysis for obtaining selected polymers dedicated to a given analyte, is the necessity of using, in the synthesis of MIP and mag-MIP, as a template the pure analyte in the amount permitting production of a required amount of the polymer [15,16]. 

Quercetin (Q), a flavonoid, multiring aromatic compound, is mainly used in food industry, medicine, in textile industry for dying of cotton and in chemical analysis. Quercetin is a flavonoid most commonly met in plants. It occurs in high concentrations in black elderberries and blueberries, close to 42 mg/100 g, in slightly lower concentrations it is found in cranberries, blackcurrants, bilberries, apricots, red grapes, cherries, blackberries, apples, strawberries, and raspberries. It should be noted that quercetin is nonuniformly distributed in particular parts of plants, e.g., the content of quercetin in unpeeled apple is close to 4.4 mg/100 g, while after peeling is drops to about 0.34 mg/100 g. Moreover, the content of quercetin increases with growing maturity of the fruit. In a bit higher concentrations is quercetin found in vegetables - its content is the highest in capers (~180.77 mg/100 g) and onion. The level of quercetin concentration depends on the species, e.g., in red onion it higher (~19.93 mg/100 g) than in yellow one (~13.27 mg/100 g), moreover its concentration is higher in the outermost scales [17,18,19].

Flowing atmospheric pressure afterglow mass spectrometry (FAPA-MS) is an analytical chemistry technique for sensitive detection of low molecular weight small organic compound. The method of analysis by mass spectrometry with the flowing atmospheric pressure afterglow ion source in the ambient atmosphere and has been proven to be a promising tool for direct and rapid determination of many compounds. The construction of the argon plasma torch and its operating parameters have a direct impact on the quality of the spectra obtained in the mass spectrometer. Argon plasma is obtained between the 2 electrodes inside the quartz tube. The first electrode is inside quartz tube and the second electrode is outside at the end of the quartz tube. The analytes were thermally desorbed and ionized by FAPA and identified in classic way by the mass spectrometer analyzer. Thermal desorption of analytes is a specific method of introducing a sample into a plasma stream. The order of release of the compounds depends on their volatility. The method offers fast and reliable structural information, with no need of pre-separation, and can be an alternative to the: EI-MS, GC-MS, ESI-MS; for fast analysis of organic compounds.

This paper presents new analytical procedures for determination of organic compounds-quercetin in real samples with the use of mag-MIP for pre-concentration and FAPA-MS with direct thermal desorption of analytes. The combination of analyte pre-concentration technique with MIP/mag-MIP and FAPA-MS analysis created new method with decreased LOD and LOQ for several trace analytes. The method proposed has been compared to that of direct determination of quercetin in water solutions based on the use of ESI-MS.

## 2. Results and Discussion 

The procedure for determination of an analyte with the use of MIP/mag-MIP by FAPA-MS comprises the following stages. 

1. Synthesis of Q-MIP/Q-mag-MIP.

2. Determination of the temperature of thermal desorption of analyte from Q-MIP/Q-mag-MIP structures. 

3. Obtaining of the empty MIP/mag-MIP with cavities permitting selective adsorption in the system structure. 

4. Selective sorption of the analyte from water solution of a studied sample on the empty MIP/mag-MIP.

5. Separation of Q-MIP/Q-mag-MIP system.

6. Determination of the analyte by FAPA-MS with thermal desorption of the analyte.

To evaluate the proposed method for quercetin determination using MIP/mag-MIP by FAPA-MS, the performance of this method was compared with quercetin determination by ESI-MS.

### 2.1. Syntheses of Q-MIP/Q-mag-MIP

At this stage of we must decide about the use of the polymers obtained, as the ability to selectively bind the analyte from the sample studied, in the conditions of competition between the analyte binding by the solvent (e.g., water) and the cavity substituents, depends on the choice of particles binding the analyte in the polymer and the character of polymerization [13]. There are two factors that have essential influence on MIP recognizability: one is steric memory-size and shape of binding cavities and the other is chemical memory-spatial arrangement of complementary functionality, both of which are correlated to the template-monomer interaction and porogen polarity. Yu and Mosbach [2] have reported that MIP for quercetin was prepared by using quercetin as template molecule and MAA or AA as functional monomer. Karaman Ersoy et al. [4] and Krnanova et al. [10] have studied the effectiveness of MIP prepared by using different functional monomers including MAA, AA, 4-vinylpyridine (4-VP), and found that the behavior of MIP towards quercetin was different in different functional monomers. Yu et al. [2], Xie et al. [5], and Song et al. [3] have prepared effective MIP for quercetin by using AA as a functional monomer. 

The polarity of the solvent, wherein MIP has been used for sorption of the analyte and the ability to form strong hydrogen bond between monomers-solvent is crucial for the choice of the synthesis method. The analyte and solvent molecules compete for the formation of bonds with terminal groups of monomers in the polymeric cavity. The quercetin molecule contains a carbonyl group and five phenolic hydroxyl groups, capable of forming hydrogen bonding interaction with amino-group of AA, carboxy-group of MAA and pyridyl-groups of 4-VP. On the other hand, in water the weakest hydrogen interaction with solvent molecules is observed for amide-groups of AA. From various MIP/mag-MIP systems, the MIP/mag-MIP obtained with the AA monomer is optimal for use in the analysis of quercetin in aqueous solutions. The basic parameter of the monomer selection was the type of interaction of its functional groups with the functional groups of the template. Acid-base properties of quercetin in aqueous solutions have been studied by Chabotarev [19] who also reported the dissociation constants (pK). The pK values assigned to the appropriate functional groups of quercetin are pK_3-OH_ = 6.4, pK_5-OH_ = 8.1, pK_7-OH_ = 9.0, pK_3′-OH_ = 9.6, pK_4′-OH_ = 11.3, respectively. Analysis of the structure-reactivity correlation allows a conclusion that the 3-OH group in the ortho position to the electron acceptor carbonyl group is the first to undergo dissociation leading to the appearance of the first anionic form in the solution. The four -OH groups participate in the formation of bonds in the polymer cavities. Isotherms of quercetin adsorption for such a system have been presented by Yu et al. [14]. As reported, the adsorption capacity increased from 267 to 1080 ng/g when the initial concentration of quercetin was increased from 2 to 14 mg/L. The value of equilibrium dissociation constants (Kd) was determined from the Scatchard plots. Kd calculated for studied materials was 2.086 mg/g and 11.76 mg/L respectively. We obtained such a system (Figure S1). 

The system Q-MIPs, obtained by adsorption of quercetin from water solution by MIPs, can be separated from the water solution by filtration or centrifugation. These processes can be difficult for polymers of low molecular mass and in electrolyte solutions. A very promising solution is based on endowment of MIPs with magnetic properties and then separation of Q-mag-MIPs by a strong magnetic field. Hybrid materials with a defined structure and function find wider application in chemical analysis. Both, MIP and mag-MIP show similar sorption properties and can be used with similar success, but mag-MIP is easier to isolate from the solution e.g., using a neodymium magnet. Magnetic hybrid materials having identical structure and properties as their non-magnetic analogues, (the same manufacturing costs) enable significant simplification and shortening of time of analytical procedures. Comparison of these two types of materials allows optimization of analytical procedures. The most commonly used molecule that is capable of endowment magnetic properties is Fe_3_O_4_. The first step to obtain mag-MIP is the preparation of magnetic nanoparticles, then the second step is to modify their surface or to functionalize the magnetic components. The third step is the polymerization in the presence of the template, functionalized monomer, and a cross-linking agent, on the surface of magnetic nanoparticles [11,13]. The process of mag-MIP synthesis is illustrated by the scheme in Figure S2.

In the determination the imprinting factor of the studied MIPs next experiments was performed. The standard solution (10 mL) of quercetin in water at an initial concentration of 10 μg mL^−1^ was mixed and incubated with 10 mg of Q-MIP, non-imprinted polymer (NIP), Q-mag-MIP, or magnetic non-imprinted polymer (mag-NIP), respectively at room temperature for 10 min. The concentration of quercetin in solution after separation of MIP/mag-MIP/NIP/mag-NIP was determined. The adsorption capacity (Q) of the quercetin to the imprinted polymers was determined using equation: Q = (C_0_ − C_E_)V/W; where C_0_ and C_E_ (mg mL^−1^) represent the initial and the equilibrium solution concentration of the adsorbed compounds, respectively, V (mL) represents the volume of the solution, and W (mg) is the weight of the imprinted polymers. The values of imprinting factor (IF) are calculated for five repetitions of measurements from the following equations: IF = Q_MIP_/Q_NIP_; where Q_MIP_ and Q_NIP_ (mg g^−1^) represent the adsorption capacity of Q-MIP and NIP, respectively. The value of IF for Q-MIP/NIP is 6.25, while for Q-mag-MIP/mag-NIP is higher and is IF = 7.75. These results indicate the higher selectivity of Q-mag-MIP versus Q-MIP and higher reliability for binding quercetin in the polymer structure. 

The products were MIP and mag-MIP capable of selective binding of quercetin from water solutions. The solubility of quercetin in water is 0.261 mg mL^−1^ [20]. 

### 2.2. Characterization of Q-MIP/Q-mag-MIP

#### 2.2.1. FTIR Spectra

Quercetin, AA monomer and NIP, and Q-MIP were subjected to FTIR analysis. A strong and broad absorption peak assigned to the stretching vibration of hydroxyl groups was found at 3300 cm^−1^. Hydroxyl groups in the quercetin molecule formed intra- and/or inter-molecular hydrogen bonds. The peak corresponding to vibrations of carbonyl group was found at 1656 cm^−1^. In addition, the peaks assigned to stretching vibration of non-aromatic skeleton (ring C) in the quercetin molecule were found at 1615 cm^−1^, 1512 cm^−1^, and 1432 cm^−1^. For AA monomer, the main absorption bands at around 3356 cm^−1^, 1673 cm^−1^, and 1613 cm^−1^ were assigned to the following vibrations: N–H stretching, being characteristic absorption peaks of primary acrylamide, C=O stretching, and C=C stretching, respectively [3,6]. The FTIR spectra of NIP/mag-NIP and Q-MIP/Q-mag-MIP were a useful tool for drawing conclusions on the structure of materials obtained (Figures S3 and S4).

For NIP and Q-MIP, the peaks at 1738 cm^-1^ and 1733 cm^−1^ (strong) were attributed to the CO stretching vibration of AA in materials, while the peak at 1678 cm^−1^ (strong) observed only for Q-MIP was attributed to the CO stretching vibration absorption from quercetin in materials. The broad peak 2830–3696 cm^−1^ was assigned to the asymmetric stretching vibration of N-H and hydrogen-bonded interaction between quercetin molecules and amid groups. For mag-NIP and Q-mag-MIP, similar signals were observed in the FTIR spectra [21]. 

#### 2.2.2. Thermal Analysis

**Figure 1:**
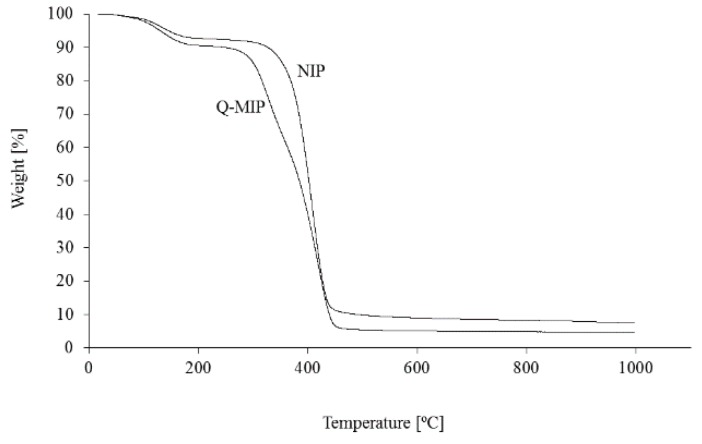
Thermogravimetric curves for Q-MIP and non-imprinted polymer (NIP).

**Figure 2:**
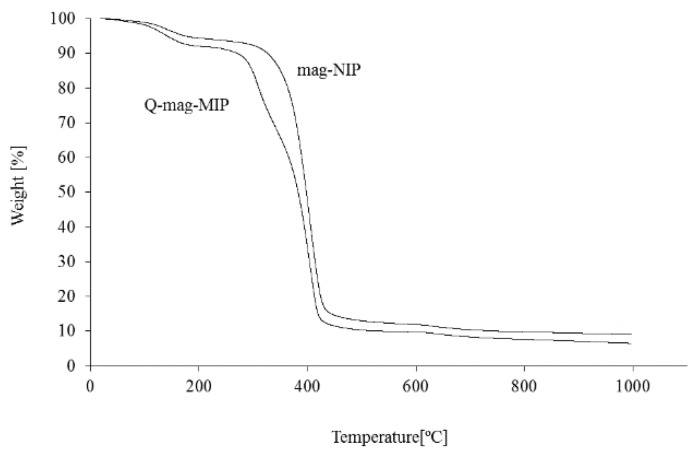
Thermogravimetric curves for Q-mag-MIP and magnetic non-molecular imprinted polymer (mag-NIP).

Thermogravimetric curves recorded for the studied materials are shown in Figure 1 and Figure 2. 

The samples Q-MIP, Q-mag-MIP, and the corresponding NIP and mag-NIP have similar thermal properties. The polymers are stable to about 250 °C, in this range a small mass loss is observed related to the loss of water from the polymer structure. The greatest mass loss is observed in the range 300–430 °C, it is more pronounced for Q-MIP and Q-mag-MIP than for NIP and mag-NIP, as it is related to the release of quercetin from the materials structures. The melting point of quercetin is 316 °C. In the range 330–350 °C, the most intensive desorption of quercetin from the samples studied is observed and in this range thermal desorption of the analyte from Q-MIP/Q-mag-MIP in FAPA-MS analysis was performed.

#### 2.2.3. SEM Images

The morphology of the synthesized samples: Q-MIP/MIP/NIP and Q-mag-MIP/mag-MIP/mag-NIP was characterized using SEM. The SEM images (low—×500 and high—×30,000 magnification) are shown in Figures S5 and S6. It is seen that the surface of MIP after the template removal is cleaner than that of polymer before the template removal of Q-MIP. 

These images reveal that the obtained mag-MIP have spherical morphology and a diameter less than 200 nm. Due to their small size, these nanoparticles agglomerate easily. 

#### 2.2.4. MS Analyzes 

a) ESI-MS

In order to establish the range of ESI-MS measurements that would permit qualitative and quantitative determination of quercetin in water solutions, in the range of negative or positive ions, a series of 10 standard water solutions of quercetin were prepared in which the concentration of quercetin varied from 10^−4^ to 10^−9^ M. The solutions were introduced to the spectrometer using a syringe pump. Measurements were made in the ranges of positive and negative ions. Exemplary spectra for 5 × 10^−4^ M concentration of quercetin in solutions are presented in Figures S7 and S8.

In the range of positive ions, the main signal is at *m*/*z* 303 [M + H]^+^, while in the range of negative ions-the one at *m*/*z* 301 [M − H]^−^. Analysis of the MS/MS^n^ spectra permitted identification of fragmentation pathways of quercetin, in the ranges of positive and negative ions (Figures S9 and S10). The fragmentation pathways are consistent with literature data [22] and are presented in Figures S11 and S12 for the range of positive and negative ions, respectively. To identify of quercetin in the samples, signals related to the molecular peak as well as to the signals the main fragmentation ions were used [23,24]. It was found that the intensity of the signal is higher for negative ions in concentration of quercetin to 5 × 10^−4^ M, however in low concentrations the signal in the positive ions begins to dominate and the changes in its intensity are more linear in relation to those in concentration. 

In order to establish the range of applicability of ESI method for determination of quercetin directly from water solutions, a series of measurements were performed. The limit of detection (MS^2^) in the range of positive ions was 10^−8^ M and in the range of negative ions it was 10^−7^ M. In ESI analysis of the lower content of quercetin in water solutions, the probability of making error in measurements or in interpretation of results is lower for the measurements in the range of positive than negative ions. Moreover, on the basis of analysis of the peak at *m*/*z* 303 (range of positive ions), the signal intensity was linearly dependent on the quercetin concentration in the quercetin solution concentration range from 10^−4^ to 10^−7^ M, which permits quantitative determination of quercetin in this range. The calculated correlation coefficient (R^2^) at 0.9999 proves the linearity of mentioned dependence (Figure S13).

Using the ESI-MS method the content of quercetin was determined in water extracts of capers and onion to be 0.00045 and 0.000045 M, which, expressed in the dry mass of capers and onion gives a quercetin content of 150 and 15 mg/100 g, respectively. 

b) FAPA-MS

**Figure 3:**
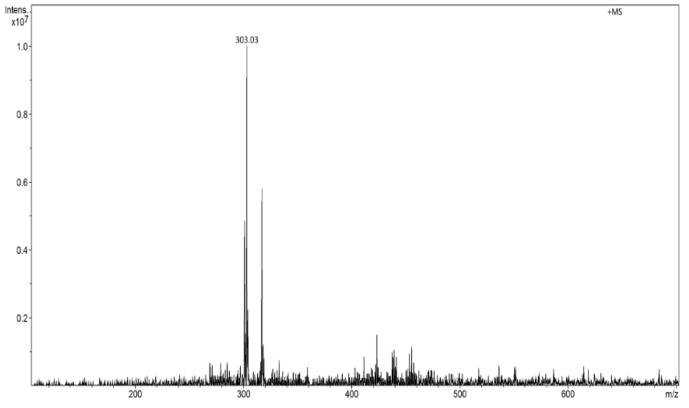
Flowing atmospheric-pressure afterglow (FAPA) spectrum of quercetin in the ranges of positive ions (*m*/*z* 303 [M + H]^+^).

The ESI-MS method was applied for quercetin determination in water solutions to get reference results that could be compared with the results obtained using MIP/mag-MIP as the selective extraction agent, analyte concentration and FAPA-MS for quantitative and qualitative analysis of the analyte. In the methods of flowing atmospheric pressure afterglow mass spectrometry, the molecules are ionized by the production and flow of thermalized ions in plasma afterglow of helium. When the sample containing the analyte is heated to 350 °C, it liberates the analyte which in the ionized form in a stream of plasma is fed to the mass spectrometer analyzer. The structure of the measuring system is presented in an earlier paper [25,26,27]. The MS spectrum of quercetin recorded in the FAPA technique shows one signal at *m*/*z* 303 in the range of positive ions (Figure 3).

**Figure 4:**
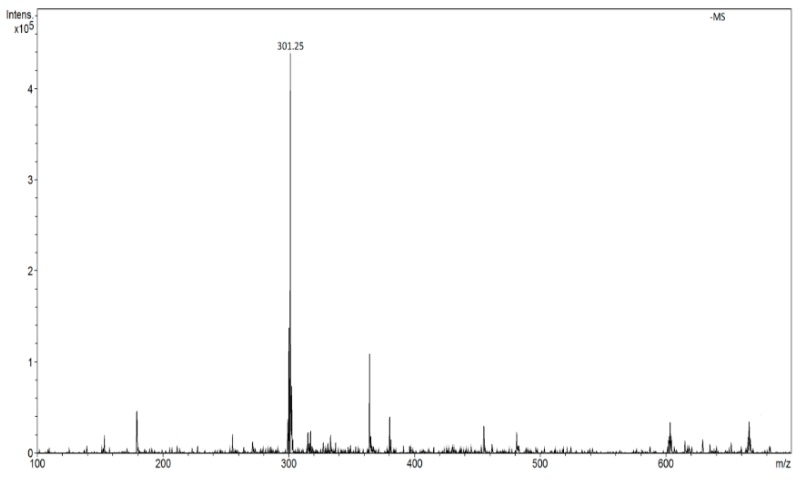
FAPA spectrum of quercetin in the ranges of negative ions (*m*/*z* 301 [M − H]^−^).

In the range of negative ions, the intensity of signals from quercetin *m*/*z* 301 is approximately 10 times smaller than that of the signal *m*/*z* 303 in positive ions (Figure 4). 

**Figure 5:**
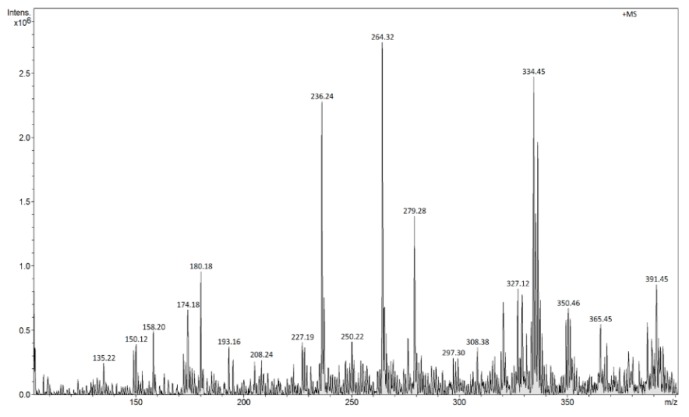
FAPA-MS spectrum of MIP in the range of positive ions.

**Figure 6:**
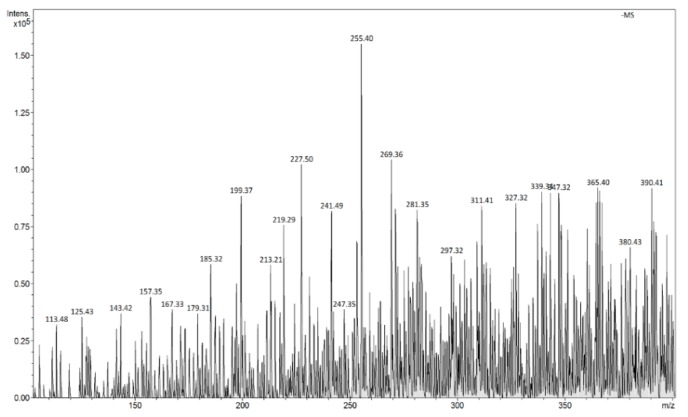
FAPA-MS spectrum of MIP in the range of negative ions.

**Figure 7:**
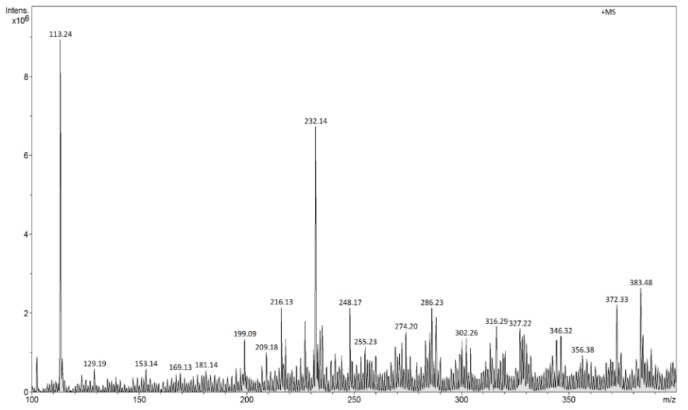
FAPA-MS spectrum of NIP in the range of positive ions.

**Figure 8:**
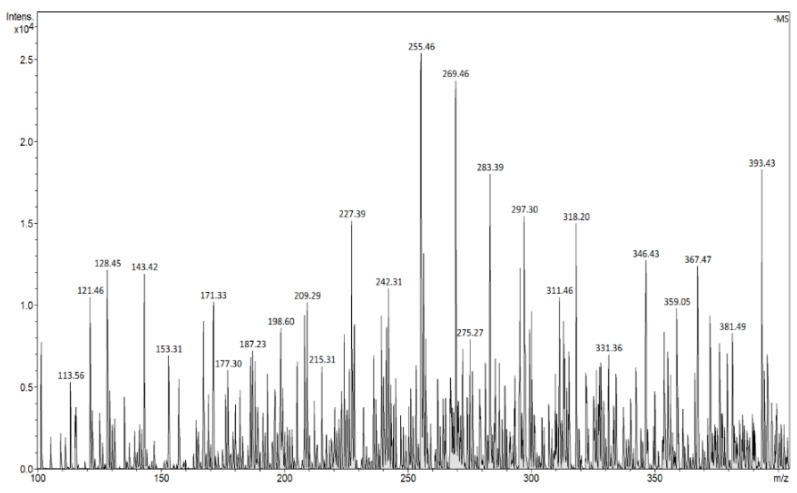
FAPA-MS spectrum of NIP in the range of negative ions.

**Figure 9:**
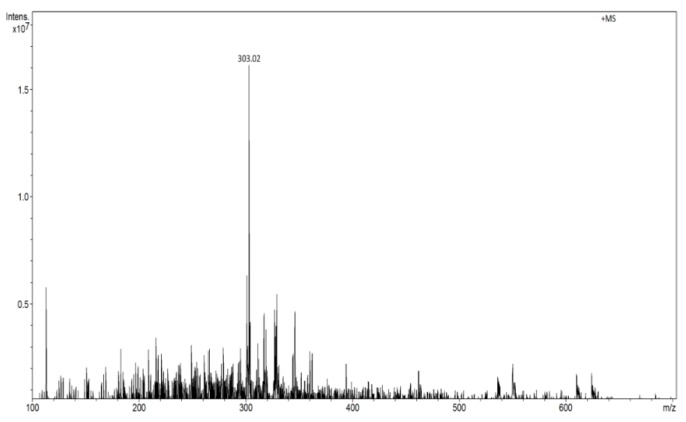
Flowing atmospheric-pressure afterglow mass spectrometry (FAPA-MS) spectrum of Q-MIP in the ranges of positive ions (*m*/*z* 303 [M + H]^+^).

**Figure 10:**
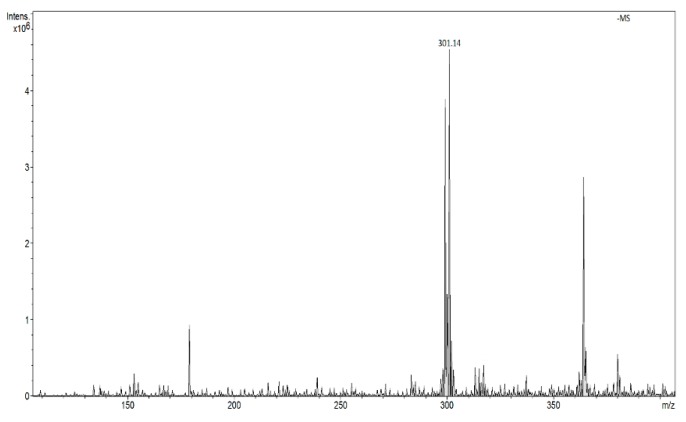
FAPA-MS spectrum of Q-MIP in the ranges of negative ions (*m*/*z* 301 [M − H]^−^).

The FAPA-MS analysis of the ions generated as a result of thermal desorption of the analyte molecules from MIP/mag-MIP (Figure 5 and Figure 6) or NIP/mag-NIP (Figure 7 and Figure 8), shows that no ions at *m*/*z* 303 positive ions and *m*/*z* 301 negative ions are generated, while the analysis of the systems containing the analyte Q-MIP/Q-mag-MIP shows that the ions at *m*/*z* 303 in the range of positive ions (Figure 9), at *m*/*z* 301 in the range of negative ions (Figure 10) characteristic of quercetin, are generated. 

The analytical results of helium plasma ionization source FAPA-MS experiments were obtained with the use of a solution of pure quercetin. Experiments were repeated three times. The relative standard deviation (RSD) obtained for all data did not exceed 15%. In order to determine the background signals, the spectra of particular matrices (NIPs) without the analytes were recorded. The limit of detection (LOD) is the concentration of a substance below which its identity cannot be distinguished from analytical artefacts. The limit of detection (LOD) was calculated according to the definition: LOD = mean blank value + 3 × standard deviation. The *m*/*z* signal of the analyte was three times higher than that of the noise level. In the experiments in which the LOD of pure compounds was determined, 20 μL of the solution of the analytes at different concentrations in methanol were introduced into the crucible and heated to about 300 °C. In the context of an analysis system, the linearity means that we assume a linear relationship between the concentration of analyte (x) and intensity of *m*/*z* signals (y), which can be written mathematically as y = f(x). It is a common practice to check the linearity of a calibration curve by inspection of the correlation coefficient (R^2^). A correlation coefficient close to unity (R^2^ = 1) is considered sufficient evidence to conclude that a perfect linear calibration has been achieved. Using different concentrations of pure analytes, different weights of the solid matrices with the analyzed compounds, it was found that the linearity of the analyte determinations was satisfied when the correlation coefficient of the function y = f(x) was greater than R^2^ = 0.9850. On the basis of measurements made for Q-MIP and Q-mag-MIP systems containing different amounts of quercetin, it was established that the signal intensity dependence on the concentration of quercetin was linear in the quercetin solution concentration range 0.015–0.075 mg Q/g MIP, while the limit of detection of the analyte in Q-MIPs was 0.005 mg/g MIP. 

The procedure for determination of quercetin in water solutions with the use of MIP/mag-MIP.

A portion of 100 mg of empty MIP, from which quercetin was removed by extraction with a solvent, should be added to 10 mL of the extract containing quercetin. After 30 min of vigorous stirring, the suspension should be centrifuged and the obtained Q-MIP system should be dried at a temperature up to 40 °C. Then the system should be placed on a heating table and analyzed by FAPA-MS. The analogous procedure with mag-MIP is similar, but the difference is that the Q-mag-MIP system is separated from the solution with the use of a neodymium magnet, which facilitated the analysis and shortened its time. 

Following this analytical procedure with MIP/mag-MIP, the content of quercetin in solutions was determined up to the concentration of 10^−9^ M. The limit of detection FAPA-MS in the range of positive ions was 10^−9^ M and in the range of negative ions it was 10^−8^ M. This concentration of quercetin solution should be assumed as the limit of quantification of the analyte by the method MIP/mag-MIP combined with FAPA-MS. 

**Figure 11:**
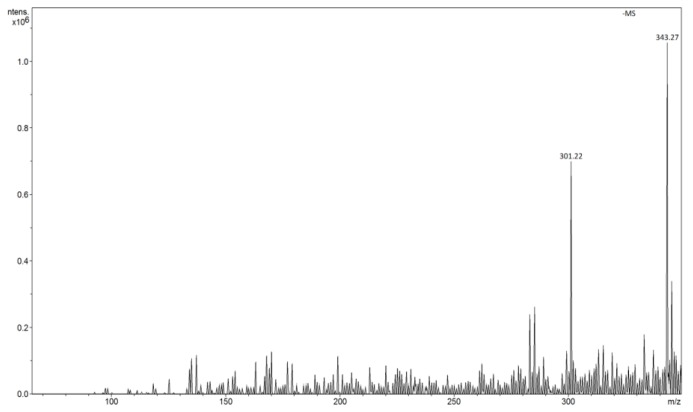
FAPA spectrum of Q-MIP after concentration of quercetin from solution of capers (*m*/*z* 301 [M − H]^−^).

**Figure 12:**
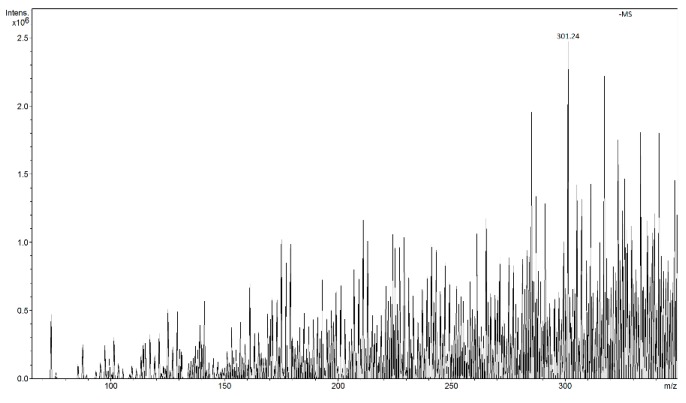
FAPA spectrum of Q-MIP after concentration of quercetin from solution of yellow onions (*m*/*z* 301 [M − H]^−^).

The method was applied for determination of quercetin in negative ions FAPA-MS, in water solutions of capers (Figure 11) and onion (Figure 12) to be of 0.00055 and 0.000049 M, which, expressed in the dry mass of capers and onion gives a quercetin content of 158 and 17 mg/100 g, respectively. The results obtained were adequate in relation to the classic ESI-MS method, but lower LOD allow of determination of compounds with lower concentrations in environmental samples. 

To comparison of two methods ESI- and FAPA-MS, the t-test was used for analysis pure quercetin in water solution and quercetin adsorbed in selective cavities in MIP polymers. This statistic test used to determine if there is a significant difference between the means of two groups, which may be related in certain features as intensity of signals *m*/*z*, LOD, the intensity of background signals and intensity of signals of ion fragmentation. For comparable of 10 parameters the two-tailed *p* value equals 0.3296. By conventional criteria, this difference is considered to be not statistically significant. Despite the lack of statistically significant difference in the results for ESI- and FAPA-MS methods, these methods fined application in the analysis of compounds in two different matrices. ESI-MS allows analyzes analytes in solution only, whereas in the FAPA-MS technique, in addition to analyzing solutions, it is possible to analyze compounds, without pre-treatment from a solid or polymer matrix.

## 3. Materials and Method

### 3.1. Materials and Chemicals 

All reagents used were commercial products. FeCl_2_·4H_2_O, FeCl_3_·6H_2_O, tetraethoxysilane (TEOS) [H226, H319, H332, H335], hydrochloric acid (HCl), citric acid, sodium hydroxide (NaOH), ammonia solution (NH_4_OH), acrylamide (AA) [H350, H340, H301, H361f, H372], ethylene glycol dimethacrylate (EGDMA) [H315, H319, H335], 2,2′-azobisisobutyronitrile solution 0.2 M in toluene (AIBN) [H242, H302, H412], Aluminum Oxide Activated, basic, Brockman I, 3-metacriloxipropiltrimetoxissilano (MPS), quercetin (Q) [H301], and all solvents (toluene [H225, H304, H336], chloroform [H302, H331, H351], *N*,*N*-Dimethylformamide (DMF) [H226, H360D, H312], ethanol, acetic acid of the purity grade p.a.), were obtained from Sigma-Aldrich (St. Louis, MO, USA).

### 3.2. Instruments

Almost all materials studied were tested by Fourier transform infrared spectroscopy and thermogravimetric method. The infrared spectra were taken on an IFS 66 *v*/*s* Fourier Transform Infrared (FTIR) spectrophotometer from Bruker (Hamburg, Germany), equipped with an MCT detector (Bruker, Hamburg, Germany, 125 scans, resolution 2 cm^−1^). The spectra were recorded in the 400–4000 cm^−1^ range for KBr pellets. In order to confirm the process of functionalization of studied materials, they were subjected to thermogravimetric analysis (TGA). The measurements were performed on a Setsys 1200 thermogravimetric analyzer (Setaram, Caluire-et-Cuire, France) with a heating rate of 10 °C min^−1^ from room temperature to 1000 °C under helium atmosphere. Scanning electron microscopy (SEM) images were acquired with QUANTA 250 FEG, FEI (Quanta, Livonia, MI, USA). 

ESI-MS and ESI-MS^*n*^ spectra were recorded using an amaZon SL ion trap (Bruker, Bremen, Germany) equipped with an electrospray ion source in infusion mode. The sample solution was introduced into the ionization source at a flow rate of 5 μL min^−1^ using a syringe pump. The apparatus was operated using the so-called “enhanced resolution mode” (mass range: 50–2200 *m*/*z*, scanning rate: 8100 *m*/*z* per second). The capillary voltage was set at −4.5 kV and the endplate offset at −500 V. The source temperature was 80 °C and the desolvation temperature was 250 °C. Helium was used as the cone gas and desolvating gas (nitrogen) at flow rates of 50 L h^−1^ and 800 L h^−1^, respectively. The mass spectrometer (Bruker, Germany) was operated in the ESI positive and negative ionization mode. In MS^*n*^ experiments, the width of the selection window was set at 2 Da and the amplification of the excitation was set according to the experiment (from 0.2 to 1.5 V). 

**Figure 13:**
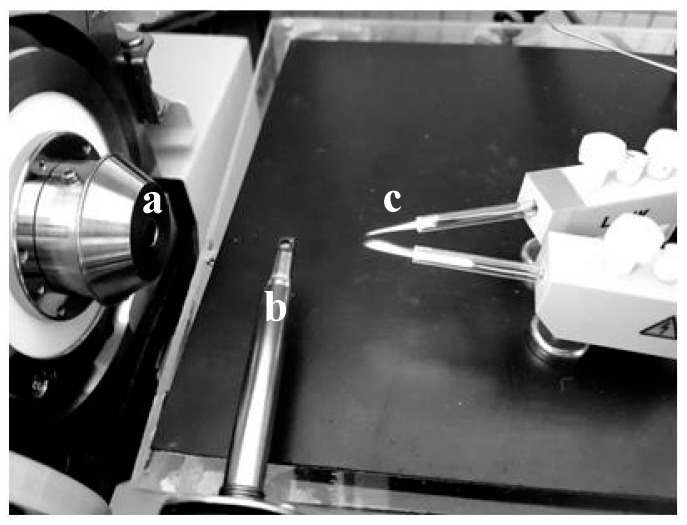
The system used to carry out the experiment: (**a**) mass spectrometer inlet, (**b**) heating system, (**c**) FAPA-ion source.

Mass spectrometers were equipped optionally in FAPA ambient plasma source (Figure 13) NOVA011 (ERTEC, Wroclaw, Poland). The experimental details for FAPA method are presented in earlier publications [26,27].

### 3.3. Synthesis 

#### 3.3.1. MIP

MIP was synthesized by the method described in literature [15]. The 1 mmol template molecule-quercetin and the 4 mmol functional monomer-AA were dissolved in a mixture of 5 ml chloroform and 5 mL DMF. Subsequently, previously purified with Aluminum Oxide Activated, basic, Brockman I, 20 mmol cross-linking agent-EGDMA were added. The ratio of the template:monomer:crosslinking agent was 1:4:20, respectively. The pre-polymerization solution was sonicated and purged with nitrogen for 40 min. Afterwards, 1.25 mL of the initiator-AIBN was added. The tube was sonicated and purged with nitrogen for additional 20 min, sealed and placed in an oven for 24 h at 60 °C. After polymerization, Q-MIP was dried under reduced pressure, and ground using mortar and pestle. The particles of quercetin were extracted with a mixture of ethanol and acetic acid (9:1, *v*:*v*) via Soxhlet extraction for 90 h. Then, the final product MIP was dried at 60 °C under vacuum and ground. 

#### 3.3.2. Mag-MIP

Mag-MIP was synthesized by the method described in literature [11]. The magnetite nanoparticles (Fe_3_O_4_) were synthesized in a two-step procedure. Firstly, magnetite (Fe_3_O_4_) was synthesized by the method described in literature [28] by using FeCl_2_·4H_2_O and FeCl_3_·6H_2_O as precursors. Secondly, the surface-functionalized magnetite was produced via hydrolysis and condensation of the TEOS organosilane agents. FeCl_2_·4H_2_O (2 g) and FeCl_3_·6H_2_O (5.2 g) were dissolved into 25 mL deoxygenated water, and then 0.85 ml of concentrated HCl were added. The resulting solution was added dropwise into 250 mL of 1.5 M NaOH solution upon vigorous stirring and N_2_ protection at 80 °C. The synthesized magnetic nanoparticles (MNPs) were separated from the solution by a powerful magnet and washed with 200 mL deionized water three times. Fe_3_O_4_ was used for the preparation of a stable aqueous suspension. A total of 10 mL of an aqueous solution of citric acid (0.5 g/mL) was added to the rigorously stirred suspension of washed nanoparticles. The pH value was set to 5.2 with a concentrated ammonia solution and heated to 80 °C. After 90 min, the pH value of the solution was elevated to 10. Lastly, the suspension was centrifuged for 5 min at 4000 rpm to remove any agglomerated nanoparticles. Next, silica coat was fabricated on the surface of Fe_3_O_4_ MNPs by the sol-gel method. 100 mL of ethanol containing 4 mL TEOS were added in the above prepared stable aqueous suspension of Fe_3_O_4_ MNPs, followed by stirring at ambient temperature for 6 h. After rinsing with ethanol and water several times, the obtained Fe_3_O_4_@SiO_2_ MNPs were dried at 60 °C. In the next step, Fe_3_O_4_@SiO_2_ was modified by MPS. For this purpose 250 mg of this material was dispersed in 50 mL of anhydrous toluene containing 5 mL of MPS and the mixture was allowed to react at 60 °C for 24 h under dry nitrogen. The mixture was filtered through a membrane, washed with toluene and dried in vacuum. The silanized material Fe_3_O_4_@SiO_2_-MPS was obtained. Then, mag-MIP was prepared by polymerization of 0.2 mmol template-quercetin and 0.8 mmol functional monomer-AA in 30 mL of a porogenic solvent-ethanol. The mixture was shaken in ultrasound bath at 25 °C for 1 h, then 200 mg of Fe_3_O_4_@SiO_2_-MPS were added into the system and the mixture was shaken for another 1 h. Furthermore, 4.0 mmol of the cross-linking agent-EGDMA and 1 mL of the initiator-AIBN were added into the system and the mixture was sonicated at 80 °C for 24 h. After polymerization, Q-mag-MIP was dried under reduced pressure, and ground using mortar and pestle. Afterwards, the template was removed after polymerization by Soxhlet extraction using a mixture of ethanol: acetic acid (9:1, *v*:*v*) as an eluent, which was replaced every 90 h. The obtained final product, mag-MIP was dried and ground. However, some water remained bound in the polymer structure. 

The level of the quercetin/template elimination was controlled by two techniques in the solution after extraction-using the ESI-MSMS technique and the FAPA-MSMS technique in dry MIPs and mag-MIPs. It was determined that after the extraction process, the materials contained about 1% and 5% of quercetin in the MIP and mag-MIP, respectively. Further extraction or solvent change did not remove the template in 100%.

#### 3.3.3. NIP and mag-NIP

As a control experiment, non-imprinted polymer (NIP) in the absence of quercetin during the polymerization was also prepared and treated in the identical manner. The magnetic non-molecular imprinted polymer (mag-NIP) was also prepared in order to compare its behavior with that of mag-MIP. The procedures were the same until the polymerization with AA, which was carried out in the absence of quercetin, unlike for mag-MIP. 

## 4. Conclusions

A new method for determination of quercetin, based on the use of MIP/mag-MIP dedicated to this analyte, was proposed. Application of the FAPA-MS method, a modern tool enabling one-step, direct testing of organic substances contained in the solid phase, allows not only their detection, but also in combination with molecularly imprinted techniques it allows development of optimal analytical methods for trace analytes determination in diluted solutions. The range of this method application was established and the results obtained using this method was compared with those achieved by the classical ESI-MS method for determination of quercetin in water solutions. On the basis of a combined use of molecular imprinting technique and the innovative FAPA-MS analytical method, a new, fast and effective method for determination of bioactive substances of low molecular mass. High-quality results in the present methods are obtained in particular for compounds of molecular mass up to 500 D as quercetin, because this technique uses the thermal desorption of analytes from a polymer matrix. The compounds not thermal desorbed significantly from polymer matrices do not ionize in the plasma stream and do not generate signals in the mass spectrometer. Compounds with higher molecular weights can be analyzed using the FAPA-MS technique by introducing the sample directly into the plasma stream. 

## Supplementary Materials

The following are available online, Figure S1: The steps for the preparation of molecularly imprinted polymer (MIP). Figure S2: General steps for the preparation of magnetic molecularly imprinted polymer (mag-MIP). Figure S3: The FTIR spectra of NIP (a) and Q-MIP (b). Figure S4: The FTIR spectra of mag-NIP (a) and Q-mag-MIP (b). Figure S5: SEM images of the Q-MIP (a,b); MIP (c,d); NIP (e,f). Figure S6: SEM images of Q-mag-MIP (a,b); mag-MIP (c,d); mag-NIP (e,f). Figure S7: ESI spectrum of quercetin in the range of positive ions (*m*/*z* 303 [M + H]^+^ and 325 [M + Na]^+^). Figure S8: ESI spectrum of quercetin in the range of negative ions (*m*/*z* 301 [M − H]^−^ and 603 [2M − H]^−^). Figure S9: ESI fragmentation spectrum of quercetin in the range of positive ion (*m*/*z* 303 [M + H]^+^). Figure S10: ESI fragmentation spectrum of quercetin in the range of negative ion (*m*/*z* 301 [M − H]^−^). Figure S11: Fragmentation pathways of quercetin in the range of positive ions. Figure S12: Fragmentation pathways of quercetin in the range of negative ions. Figure S13: Intensity of the positive signal at *m*/*z* 303 versus quercetin concentration in solution (M).

## References

[1] Pakade V.E., Molefe E.D., Tavengwa N.T. (2017). Quantitative determination of trace concentrations of quercetin from prickly pear skin complex sample extracts by application of molecularly imprinted polymers. J. Environ. Chem. Eng..

[2] Yu C., Mosbach K. (1997). Molecular Imprinting Utilizing an Amide Functional Group for Hydrogen Bonding Leading to Highly Efficient Polymers. J. Org. Chem..

[3] Song X., Li J., Wang J., Chen L. (2009). Quercetin molecularly imprinted polymers: Preparation, recognition characteristic and properties as sorbent for solid-phase extraction. Talanta.

[4] Ersoy S.K., Tutem E., Baskan K.S., Apak R., Nergiz C. (2016). Preparation, characterization and usage of molecularly imprinted polymer for the isolation of quercetin from hydrolyzed nettle extract. J. Chromatogr. B.

[5] Xie J., Zhu L., Luo H., Zhou L., Li C., Xu X. (2001). Direct extraction of specific pharmacophoric flavonoids from gingko leaves using a molecularly imprinted polymer for quercetin. J. Chromatogr. A.

[6] Curcio M., Cirillo G., Parisi Q.I., Iemma F., Picci N., Puoci F. (2012). Quercetin-Imprinted Nanospheres as Novel Drug Delivery Devices. JFB.

[7] Hong Y., Chen L. (2013). Extraction of quercetin from Herba Lysimachiae by molecularly imprinted-matrix solid phase dispersion. J. Chromatogr. B.

[8] Kudrinskaya V.A., Dmitrienko S.G., Zolotov A. (2009). Synthesis and Study of Sorption Properties of Molecularly Imprinted Polymers for Quercetin. Moscow Univ. Chem. Bullet..

[9] Pardo A., Mespouille L., Blankert B., Trouillas P., Surinf M., Dubois P., Duez P. (2014). Quercetin-imprinted chromatographic sorbents revisited: Optimization of synthesis and rebinding protocols for application to natural resources. J. Chromatogr. A.

[10] Krňanová J., Denderz N., Lehotay J., amohýl M. (2015). Determination of Some Flavonoids by HPLC Using Quercetin-Molecularly Imprinted Polymers. J. Liquid Chromatogr. Related Technolo..

[11] Uzuriaga-Sánchez R.J., Khan S., Wonga A., Picasso G., Pividori M.I., Taboada Sotomayor M.D.P. (2016). Magnetically separable polymer (Mag-MIP) for selective analysis of biotin in food samples. Food Chem..

[12] Marć M., Kupka T., Wieczorek P.P., Namieśnik J. (2018). Computational modeling of molecularly imprinted polymers as a green approach to the development of novel analytical sorbents. Trends Analy. Chem..

[13] Guć M., Schroeder G. (2017). The Molecularly Imprinted Polymers. Influence of Monomers on The Properties of Polymers-A Review. World J. Res. Rev. (WJRR).

[14] Yu L., Yun Y., Zhang W., Wang L. (2011). Preparation, recognition characteristics and properties for quercetin molecularly imprinted polymers. J. Desalination Water Treatment.

[15] del Mar Castro López M., Cela Pérez M.C., Dopico García M.S., López Vilarino J.M., González Rodríguez M.V., Barral Losada L.F. (2012). Preparation, evaluation and characterization of quercetin-molecularly imprinted polymer for preconcentration and clean-up of catechins. Analy. Chim. Acta.

[16] Pakade V., Lindahl S., Chimuka L., Turner C., Dmitrienko S.G., Kudrinskaya V.A., Apyari V.V. (2012). Methods of Extraction, Preconcentration, and Determination of Quercetin. J. Analy. Chem..

[17] Kobylińska A., Janas K.M. (2015). Prozdrowotna rola kwercetyny obecnej w diecie człowieka. Postepy. Hig. Med. Dosw..

[18] Gheribi E. (2011). Związki polifenolowe w owocach i warzywach. Medycyna Rodzinna.

[19] Chebotarev A.N., Snigur D.V. (2015). Study of the Acid Base Properties of Quercetin in Aqueous Solutions by Color Measurements. J. Analy. Chem..

[20] Quercetin. https://www.drugbank.ca/drugs/DB04216.

[21] Scigelova M., Hornshaw M., Giannakopulos A., Makarov A. (2011). Fourier Transform Mass Spectrometry. Mol. Cell. Proteomics.

[22] Zenkevich I.G., Eshchenko A.Y., Makarova S.V., Vitenberg A.G., Dobryakov Y.G., Utsal V.A. (2007). Identification of the Products of Oxidation of Quercetin by Air Oxygen at Ambient Temperature. Molecules.

[23] Tiberti L.A., Yariwake J.H., Ndjokob K., Hostettmannb K. (2007). On-Line LC/UV/MS Analysis of Flavonols in the Three Apple Varieties Most Widely Cultivated in Brazil. J. Braz. Chem. Soc..

[24] Fabre N., de Pharmacognosie I.R.L., de Hoffmann E., Quetin-Leclercq J. (2001). Determination of Flavone, Flavonol, and Flavanone Aglycones by Negative Ion Liquid Chromatography Electrospray Ion Trap Mass Spectrometry. J. Am. Soc. Mass Spectrum..

[25] Smoluch M., Reszke E., Ramsza A., Labuz K., Silberring J. (2012). Direct analysis of methcathinone from crude reaction mixture by flowing atmospheric-pressure afterglow mass spectrometry. Rapid Commun. Mass Spectrom..

[26] Ceglowski M., Kurczewska J., Smoluch M., Reszke E., Silberring J., Schroeder G. (2015). Magnetic scavengers as carriers of analytes for flowing atmospheric pressure afterglow mass spectrometry (FAPA-MS). Analyst.

[27] Cegłowski M., Smoluch M., Reszke E., Silberring J., Schroeder G. (2015). Flowing atmospheric pressure afterglow combined with laser ablation for direct analysis of compounds separated by thin-layer chromatography. Anal. Bioanal. Chem..

[28] Ghafoor S., Ata S. (2017). Synthesis of carboxyl-modified Fe_3_O_4_@SiO_2_ nanoparticles and their utilization for the remediation of cadmium and nickel from aqueous solution. J. Chil. Chem. Soc..

