# Supplementary material for: Application of Molecularly Imprinted Polymers (MIP) and Magnetic Molecularly Imprinted Polymers (mag-MIP) to Selective Analysis of Quercetin in Flowing Atmospheric-Pressure Afterglow Mass Spectrometry (FAPA-MS) and in Electrospray Ionization Mass Spectrometry (ESI-MS)
Source: Molecules. 2019 Jun 26;24(13):2364. doi: 10.3390/molecules24132364 (PMC6651046; doi:10.3390/molecules24132364)
Supplement: Supplementary file 1 [file molecules-24-02364-s001.pdf]

Supplementary Material

Application of molecularly imprinted polymers (MIP) and magnetic molecularly imprinted polymers (mag-MIP) to selective analysis of quercetin in flowing atmospheric-pressure afterglow mass spectrometry (FAPA-MS) and in electrospray ionization mass spectrometry (ESI-MS)

Maria Guć and Grzegorz Schroeder

Faculty of Chemistry, Adam Mickiewicz University in Poznań, Uniwersytetu Poznańskiego 8, 61-614 Poznań, Poland

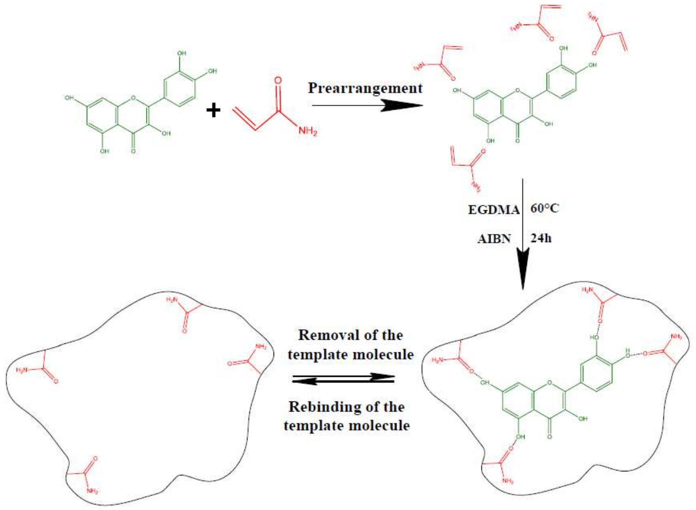

Figure S1. The steps for the preparation of molecularly imprinted polymer (MIP).

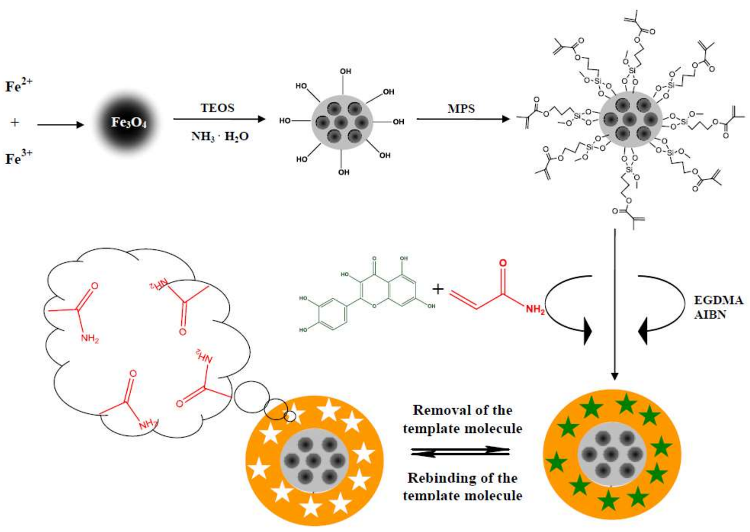

Figure S2. General steps for the preparation of magnetic molecularly imprinted polymer (mag-MIP).

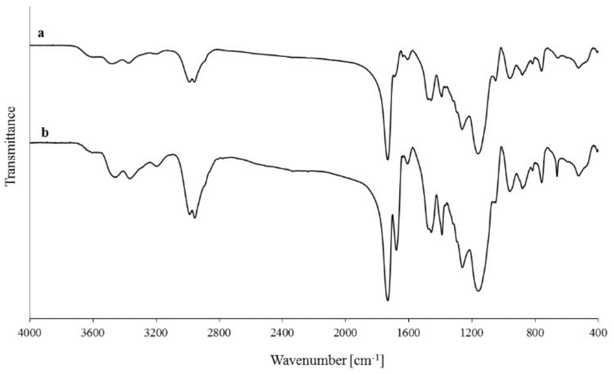

Figure S3. The FTIR spectra of NIP (a) and Q-MIP (b).

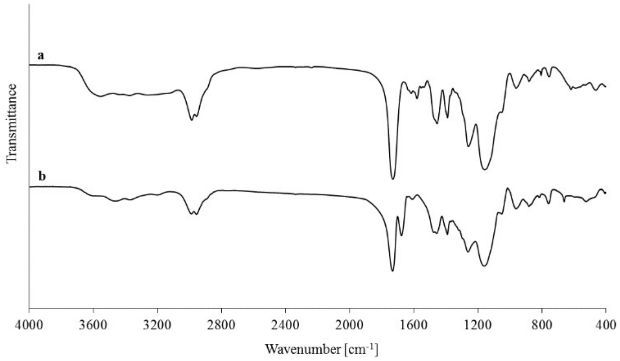

Figure S4. The FTIR spectra of mag-NIP (a) and Q-mag-MIP (b).

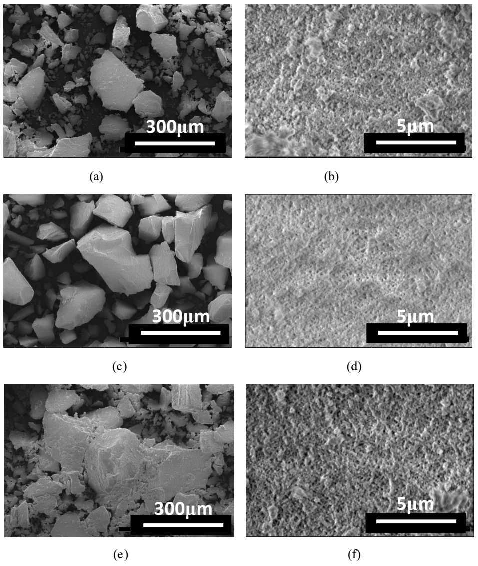

Figure S5. SEM images of the Q-MIP (a,b); MIP (c,d); NIP (e,f).

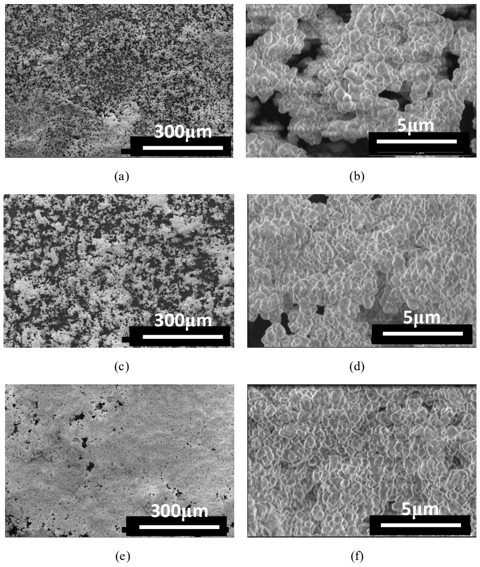

Figure S6. SEM images of Q-mag-MIP (a,b); mag-MIP (c,d); mag-NIP (e,f).

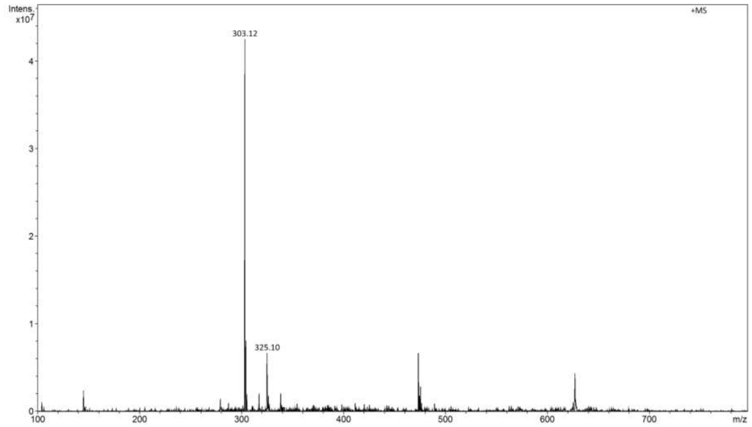

Figure S7. ESI spectrum of quercetin in the range of positive ions (m/z 303 $[M+H]^+$ and 325 $[M+Na]^+$).

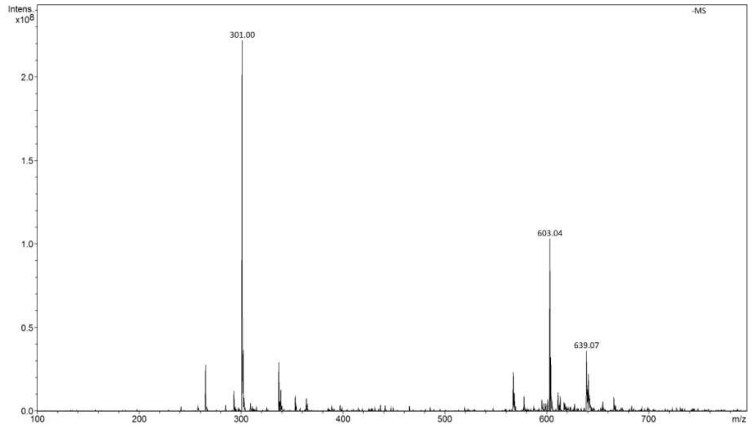

Figure S8. ESI spectrum of quercetin in the range of negative ions (m/z 301 $[M-H]^-$ and 603 $[2M-H]^-$).

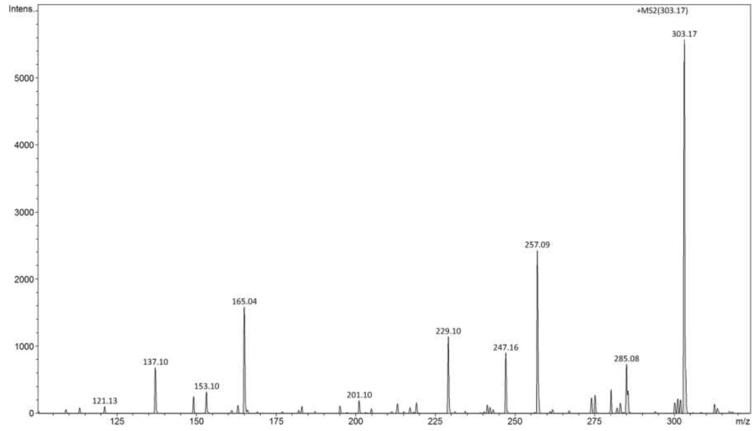

Figure S9. ESI fragmentation spectrum of quercetin in the range of positive ion (m/z 303 [M+H]$^+$).

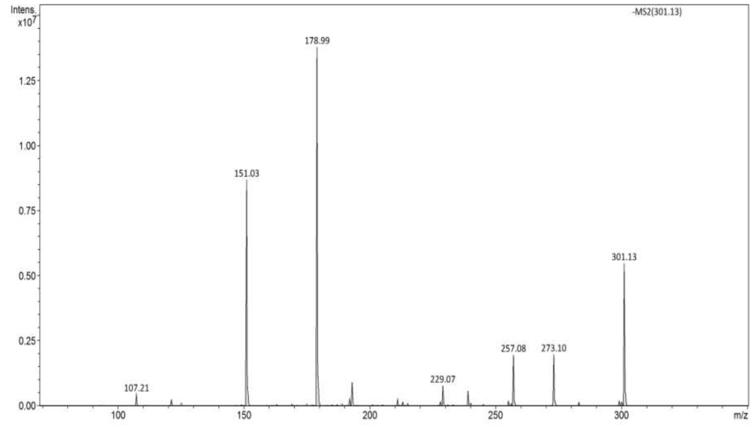

Figure S10. ESI fragmentation spectrum of quercetin in the range of negative ion (m/z 301 [M-H]$^-$).

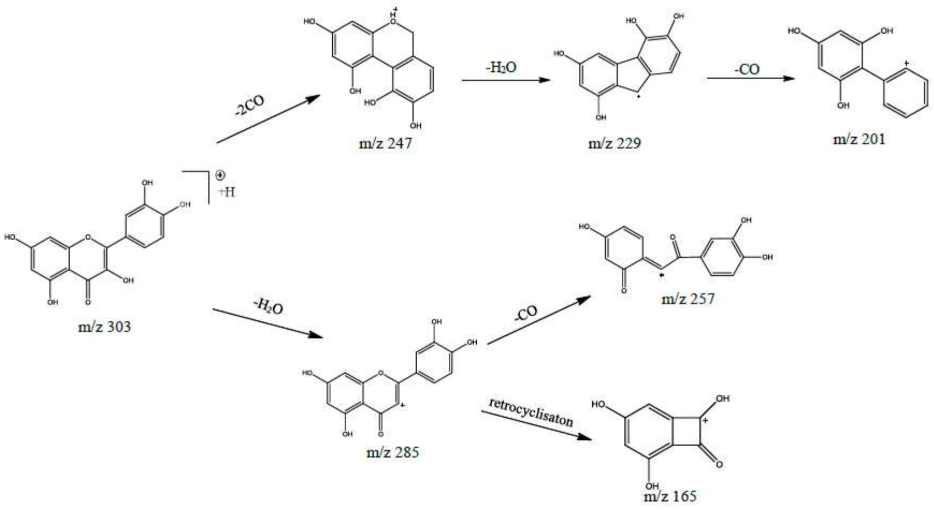

Figure S11. Fragmentation pathways of quercetin in the range of positive ions.

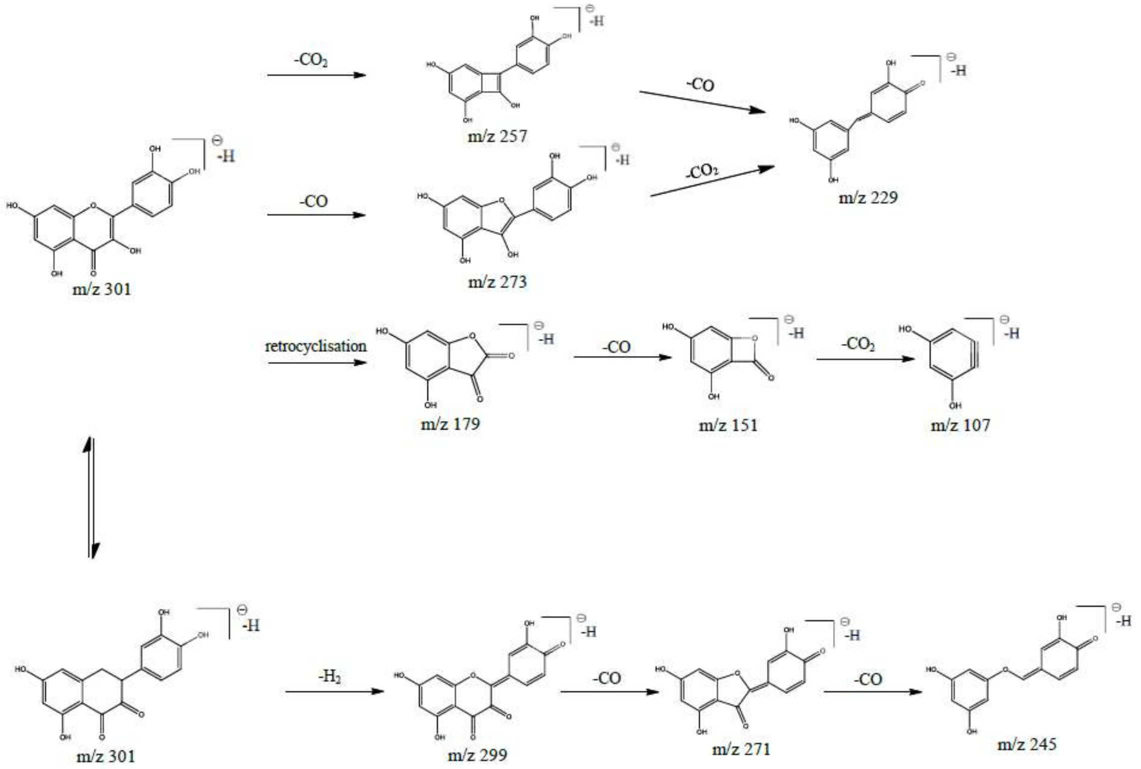

Figure S12. Fragmentation pathways of quercetin in the range of negative ions.

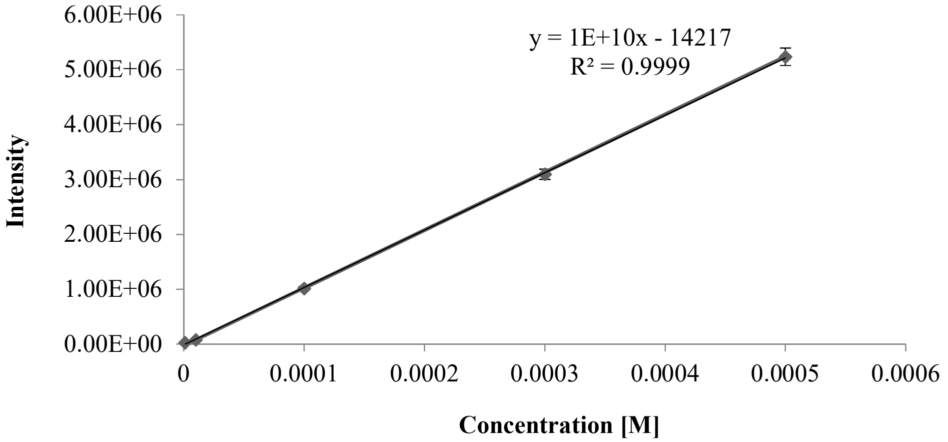

Figure S13. Intensity of the positive signal at m/z 303 versus quercetin concentration in solution (M).
